# Prevalence and associated factors of active trachoma among children aged 1-9 years old in mass drug administration graduated and non-graduated districts in Northwest Amhara region, Ethiopia: A comparative cross-sectional study

**DOI:** 10.1371/journal.pone.0243863

**Published:** 2020-12-15

**Authors:** Gashaw Melkie, Muluken Azage, Genet Gedamu

**Affiliations:** 1 Department of Environmental Health, School of Public Health, Teda Health Science College, Gondar, Ethiopia; 2 Department of Environmental Health, School of Public Health, College of Medicine and Health Sciences, Bahir Dar University, Bahir Dar, Ethiopia; University of Pretoria, SOUTH AFRICA

## Abstract

**Background:**

Mass drug administration has implemented to reduce trachoma since 2001, however, trachoma is still the major public health problem in Amhara Region, Ethiopia. However, credible evidence on the prevalence of trachoma and its associated factors after the implementation of mass drug administration is limited.

**Objective:**

To assess the prevalence and associated factors of active trachoma among children aged 1–9 years old in mass drug administration graduated and non-graduated districts in the Northwest Amhara Region.

**Methods:**

A comparative cross-sectional study was conducted from October to November, 2019. A stratified multistage random sampling was used to select 690 households having children aged 1–9 years. Data were collected using a pretested structured questionnaire. Data were entered into Epi-data version 3.1 and exported to SPSS version 20.0 for analysis. Bivariate and multivariable logistic regressions were employed to identify factors associated with active trachoma. Crude and adjusted odds ratios with 95% confidence interval were computed to assess the degree of association between the independent variables and active trachoma.

**Results:**

The overall prevalence of active trachoma was 8.3% (95% CI: 6.2% –10.5%) and showed a significant variation between graduated [3.5% (95% CI: 1.8% –5.6%)] and non-graduated [13% (95% CI: 9.7%–16.8%)] districts. Living in graduated districts (AOR = 7.39, 95% CI: 3.19, 17.09), fly presence in the house (AOR = 3.14, 95% CI: 1.43, 6.89), presence of more than two children in the family (AOR = 3.78, 95%CI: 1.79, 7.98), did not wash face daily (AOR = 6.31, 95% CI: 1.81, 21.98), did not use soap during face washing (AOR = 3.34, 95% CI: 1.37, 8.15), presence of sleep in eyes (AOR = 3.16, 95% CI: 1.42, 7.02) and presence of dirt on child face (AOR = 2.44, 95% CI: 1.08, 5.50) increased the odds of having active trachoma.

**Conclusion:**

The prevalence of active trachoma was high in the study area and showed a significant variation between graduated and non-graduated districts with mass drug administration. Living in non-graduated districts, fly presence in the house, more than two children in a household, did not wash the face daily, did not use soap during face washing, presence of sleep in eyes, and dirt on the child’s face were the significant predictors of active trachoma. Therefore, the identified modifiable factors are the area of intervention to reduce the burden of active trachoma.

## Introduction

Trachoma is one of the common infectious diseases that cause blindness due to recurrent ocular infection with *Chlamydia trachomati*s in the world [1, 2]. Children are the main reservoir of C. trachomatis infection [3]. It is a public health problem in 44 countries and 137 million people living in trachoma endemic areas who are at risk of trachoma blindness [4]. It is responsible for the blindness or visual impairment of about 1.9 million people and trachomatous visual loss yields 39 million lifetimes Disability-Adjusted Life Years (DALYs) [5]. Besides, it also causes the potential productivity loss of 2.9 billion dollars per year [5].

Five countries namely Ethiopia, India, Nigeria, Sudan, and Guinea account for 48.5% global burden of active trachoma, and three countries namely China, Ethiopia, and Sudan account for 50% of the global burden of trichiasis [6].

In Ethiopia, trachoma is the second major cause of blindness and the third major cause of low vision [7]. The prevalence of active trachoma among children aged 1–9 years old was 40.1% and it is widely distributed in the country. The highest prevalence was registered in the Amhara region (62.6%) which is higher compared to other regions such as Oromia (41.3%), and Southern Nations, Nationalities, and Peoples (SNNP) (33.2%) [8]. In the Amhara region, impact surveys in 2015 reported the average prevalence of active trachoma was 25.9% (24.9%–26.9%) [9].

Socio-demographic factors such as increased family size and the number of children aged 1–9 years old in the household that creates crowded living conditions increase the transmission of active trachoma [10]. Childhood hygiene behaviors such as ocular and nasal secretions and unclean faces attract flies and provides a vehicle for transmission [11]. Similarly, water scarcity also promotes the transmission which in turn compromises hygienic practices, like face washing. Limited access to latrines increases fecal contamination of the environment which favors fly breading is also a mechanical vector in trachoma transmission [12–15].

Mass Drug Administration (MDA) with prevention of risk factors; Surgery, Antibiotics, Facial cleanliness and Environmental improvement (SAFE) have been the focus areas of intervention in endemic countries, including Ethiopia [16, 17]. The intervention was introduced in the Amhara region in 2001 and expanded to both graduated and non-graduated districts in the current study area in 2007 [9]. As a program success, the graduated district included in this study discontinued the intervention in 2016 by declaring less than 5% prevalence of active trachoma among children aged 1–9 years old. Whereas, other endemic districts (non-graduated) are still struggling to be free of active trachoma [18]. However, to the best of our knowledge, there is no evidence whether graduated districts sustain less than 5% prevalence of active trachoma after stopping MDA. The prevalence of active trachoma has not been well investigated between graduated and non-graduated districts with MDA. Therefore, this study aimed to assess the prevalence and associated factors of active trachoma among children aged 1–9 years old in mass drug administration graduated and non-graduated districts in Central Gondar Zone, Amhara Region, Northwest Ethiopia.

## Methods and materials

### Study settings and design

A community-based comparative cross-sectional study was conducted in two previously trachoma-endemic districts of Central Gondar Zone, Amhara Region, Ethiopia from October 1, 2019, to November 15, 2019. Gondar is the zonal city town that is located at 750 km in the Northwest direction of Addis Ababa, the capital city of Ethiopia, and 175 km from Bahir Dar, the capital city of Amhara National Regional State. The Zone has one urban and 14 rural districts. Of the rural districts, 8 have not achieved < 5% Trachomatous inflammation -follicular (TF) which are considered as non-graduated districts, and the rest districts are graduated. The non-graduated districts are receiving azithromycin every year to achieve <5% TF [18].

### Study participants

All children age 1–9 years in Central Gondar Zone were the source population of the study. The study participants were children aged 1–9 years in selected households (HHs) in the study area. **Eligibility criteria**. All mothers/caregivers who lived in eligible HHs were included in the interview. All children aged 1–9 years old live in the study area during the study period were included.

All mothers/caregivers from eligible HHs who are unable to hear and speak in the study area were excluded from the interview. Children who are blind and who had serious medical sickness were excluded from the study.

### Sample size determinations

The sample size was determined using two population proportions formula using Epi–info software with the following assumptions; 95% confidence interval, the proportion of active trachoma in controls (P_1_ = 31.8%) and cases (P_2_ = 48.0%) [19], 1 to 1 ratio in graduate and non-graduate districts, design effect 2 and 10% non-response rate. The final adequate sample size was 690 HHs (345 from graduated and 345 non-graduated districts) having children aged 1–9 years old.

### Sampling technique and procedure

A multi-stage stratified sampling technique was used to select HHs having children aged 1–9 years old in MDA graduated and non-graduated districts. Stratification of the districts was done based on the presence and absence of a yearly MDA campaign in the district. Two districts, one district from MDA graduated districts(Lay Armacheho) and Wegera from the non-graduated districts, were selected randomly. Eight kebeles (lowest administrative unit in Ethiopia) from each selected district, 22% of the total kebeles of each district, were randomly selected. Proportion to size allocation was made to determine the required sample size for each randomly selected district and kebele. Households that have children aged 1–9 years old were selected using a systematic random sampling technique. To determine the interval of HH in selected Kebele, K^th^ value was used. If there were two or more children in a single HH, one child was selected using a simple random sampling technique.

### Data collection tools

Data were collected using a structured questionnaire, observation, and physical examination. The questionnaire was developed in English then translated into Amharic (local language) and then back to English to ensure consistency. Interviewing was conducted by two environmental health professionals, physical examination and grading of trachoma was done by tained and certified nurses, and two supervisors were assigned to manage data collection process. Grading of trachoma was done by assessing diagnostic signs using 2.5X loupes as recommended elsewhere [20]. Environmental health professionals documented the presence of any ocular or nasal secretions and any fly-eye contact during the minutes before the clinical examination. Both eyes were examined for signs of active trachoma (TF) using the WHO Trachoma Grading System [20]. A child was reported as positive for active trachoma if he/she had the signs and symptoms of active trachoma (TF) in either of his/her eyes.

### Study variables

The dependent variable was active trachoma among children aged 1–9 years old. Independent variable included socio-demographic variables (Maternal and paternal occupation, marital status, level of education of father and mother, age of the child, sex of the child, household wealth index, household size and number of children aged 1–9 years old); Environmental and household factors (source of water, time taken to fetch water, quantity of water used daily, availability of latrine, type of latrine, latrine utilization, presence of feces in the house compound, flies in the house, waste disposal sites, availability of livestock, livestock in the house and overcrowding status); Child’s behavioral factors (Frequency of face washing (determined by asking the mother or caregivers), using soap for washing (determined by asking themother or caregivers), discharge on the eye, discharge from nose, facial cleanness and presence of flies on child face); Knowledge about trachoma (Knowledge on mode of transmission and knowledge on how to prevent trachoma); and Prevention and control of chlamydia trachomatis infection (Frequency of *Zithromax* prophylaxes dose received, health education about trachoma).

### Operational definitions

#### Active trachoma

The presence of Trachomatous inflammation follicular (TF) in either of the child’s eyes, the WHO’s criteria/indicator for the elimination of trachoma [21].

#### Trachomatous inflammation -follicular (TF)

The presence of five or more follicles in the upper tarsal conjunctiva each at least 0.5 mm in size [20].

#### Facial cleanliness

The absence of ocular discharge, nasal discharge, flies on the face, fly-eye contacts, and/or any dirt on the face at the time of clinical examination [22, 23].

#### Any dirt on the child’s face

The presence of any dust and food on the face at the time of clinical examination.

#### Sleep in the eyes

The eye discharge collects and crusts in the corners of the eyes and sometimes along the lash line.

#### Improved water source

The design and nature of its construction, adequately protects the water from outside contamination, in particular from fecal matter, and includes piped water systems, hand pumps, public tap/standpipe, protected well/spring otherwise it is ‘Un-improved water source’.

#### Improved sanitation facility

Sanitation facility that hygienically separates human excreta from human contact [24] which includes pour-flush latrine, Ventilated improved pit latrine (VIP), pit latrine with slab otherwise ‘Unimproved sanitation type’.

#### Proper solid waste disposal

Solid waste disposal method that separates household generated solid waste hygienically which includes solid waste in the pit prepared for it, burning near the yard, collected and disposed of by the municipality.

#### Graduated district

A district achieved <5% TF prevalence among children aged 1–9 years old after successive mass azithromycin treatment and stopped yearly MDA campaign.

#### Non-graduated district

A district that is not achieving <5% TF prevalence among children aged 1–9 years old after successive mass azithromycin treatment and is under the implementation of the MDA campaign.

#### Knowledge about trachoma

Mothers/caregivers of children were asked about the signs and symptoms, modes of transmission, and prevention methods of trachoma. Their responses are categorized as good knowledgeable who scored more than 80% questions correctly, fairly knowledgeable who scored 50% to 79.9% questions correctly, and less knowledgeable who scored less than 50% questions correctly.

### Data quality control

Training was given for data collectors and supervisors on the purpose of the study, data collection technique, and tool by the principal investigator for two days. Pre-test of the questionnaires on 5% of the sample size on 30 households in Kebeles where the study was not undertaken. The pre-test was part of the training and its findings were discussed during the training day and all the concerns were clarified. Every day after data collection, filled questionnaires were reviewed by supervisors and the principal investigator for ensuring completeness of questions. Incomplete questionnaires were discarded from the analysis.

### Ethical considerations

Ethical approval was obtained from the Institutional Review Board of Bahir Dar University College of Medicine and Health Sciences. Permissions letter was also taken from Amhara Public Health Institute (APHI), Central Gondar Zonal Health Department, and Lay-Armacheho and Wegera district health offices. Informed verbal consent and assent were obtained from the sampled children’s parents. The respondents were also informed that they have the full right to withdraw or refuse at any time from the process. Confidentiality of information given by each respondent was kept properly and anonymity was explained clearly for the participants. Children diagnosed with active trachoma were referred to the nearby health center for further investigation and treatment.

### Data analysis

Data were entered into Epi-Data version 3.1 and exported to SPSS statistical package version 20.0 for analysis. Descriptive statistics were used to describe the data. The principal component analysis was computed to calculate the wealth status of HHs. Bivariate and multivariable logistic regressions were used to identify predictors of active trachoma. The Hosmer-Lemeshow test was checked to assess the model fitness(p-value 0.816). A p-value < 0.25 was used as criteria during bivariate analysis to retain variables for the multivariable logistic regression model. The backward stepwise logistic regression model was used to control multi-collinearity and confounding effects. Multicollinearity was checked using variance inflation factors (VIF). Crude and adjusted odds ratios with 95% confidence interval were calculated to measure the degree of association between independent variables and active trachoma. A p-value < 0.05 was considered as a level of statistical significance.

## Results

### Socio-demographic characteristics

A total of 678 children aged 1–9 years old, 339 from the MDA graduated district, and 339 from the MDA non-graduated district, was participated in the study with a response rate of 98.3%. Nearly half (51.8%) of all the study participants were males with 53.8% and 50.1% in graduated and non-graduated districts, respectively. The mean age (± SD) of children in MDA graduated and non-graduated districts were comparable, 4.65 (± 2.47) and (4.96 (± 2.51) years, respectively. The majority of children (87%) in MDA graduated and (82.3%) in non-graduated districts were from a family with 1–2 children aged 1–9 years old. More than two-thirds of children (69.9%) in MDA graduated and (70.8%) of non-graduated districts were not attending school. Regarding mother’s educational status, 81.1% and 85.0% of mothers in MDA graduated and non-graduated districts, respectively, had no formal education whereas only 13.3% of mothers in MDA graduated and 5.9% of mothers in the non-graduated district, attended secondary level education and above. The mean family size of (± SD) household was 4.67 (±1.49) and 5 (±1.63)in MDA graduated and non-graduated districts, respectively. The mean number of children aged 1–9 years old(± SD) in a given household was 1.79 (± 0.68) and 1.78 (± 0.91) in MDA graduated and non-graduated districts, respectively (Table 1).

**Table 1:** Socio-demographic characteristics of participants in mass drug administration graduated and non-graduated district, Central Gondar, Ethiopia, December 2019.

Variables	Graduated		Non-graduated		Total		Chi-square (p-value)
	Frequency	(%)	Frequency	(%)	Frequency	(%)	
Child age group in Years							.377
1–3	128	37.8	119	35.1	247	36.4	
4–6	116	34.2	109	32.2	225	33.2	
7–9	95	28	111	32.7	206	30.4	
Sex of the child							.404
Male	181	53.4	170	50.1	351	51.8	
Female	158	46.6	169	49.9	327	48.2	
Family size of the HH							< .0001
1–5	246	72.6	232	68.4	478	70.5	
≥ 6	93	27.4	107	31.6	200	29.5	
No. of children aged1-9 years							< .0001
1–2	295	87	279	82.3	574	84.7	
≥ 3	44	13	60	17.7	104	15.3	
Children share the same sleeping space							.004
Yes	205	60.5	155	45.7	360	53.1	
No	134	39.5	184	54.3	318	49.9	
Maternal Education							.048
No formal education	275	81.1	288	85.0	563	83.0	
Primary level	19	5.6	31	9.1	50	7.4	
≥ Secondary level	45	13.3	20	5.9	65	9.6	
Maternal occupation							.715
Unemployed	43	12.7	8	2.4	51	7.5	
House wife	241	71.1	264	77.9	505	74.5	
Others	55	16.2	67	19.8	122	18.0	
Marital status							.516
Single	-	-	7	2.1	7	1.0	
Married	290	85.5	300	88.5	590	87.0	
Divorced	32	9.4	17	5.0	49	7.2	
Widowed	5	1.5	7	2.1	12	1.8	
Lives separately	12	3.5	8	2.4	20	2.9	
Relation with the Child							.987
Parent	333	98.2	309	91.2	642	94.7	
Guardian	6	1.8	30	8.8	36	5.3	
Child Education							.427
Not yet Attended	237	69.9	240	70.8	477	70.4	
Attended	102	30.1	99	29.2	201	29.6	
Husband Education							.041
No formal education	240	79.5	223	72.4	463	75.9	
Primary level	24	7.9	58	18.8	82	13.4	
Secondary and above	38	12.6	27	8.8	65	10.7	
Wealth index							.385
Highest	134	39.5	92	27.1	226	33.3	
Middle	135	39.8	91	26.8	226	33.3	
Lowest	70	20.6	156	46.0	226	33.3	
Place of residence							.339
Urban	69	20.4	47	13.9	116	17.1	
Rural	270	79.6	292	86.1	562	82.9	

### Environmental and housing condition of the households

Among all households in the graduated district, 199 (58.7%) had an improved water source, 200 (59.0%) took more than 30 minutes to fetch water from the water source. Of all households in a non-graduated district, 328 (96.8%) had an improved water source and 124 (36.6%) took more than 30 minutes to fetch water from the source. The average daily water consumption was found to be 12.94 liters/person/day and 13.57 liters/person/day for a household in graduated and non-graduated districts, respectively. Among selected households, 243 (71.7%) in graduated and 232 (68.4%) in non-graduated districts had latrine of any type. Of these, 240 (70.8%) and 206 (60.8%) households in graduated and non-graduated districts utilized their latrine, respectively (Table 2).

**Table 2:** Environmental and housing condition of households in mass drug administration graduated and non-graduated district, Central Gondar, Ethiopia, December 2019.

Variables	Districts				Total		Chi-square (p-value)
	Graduated		No-graduated				
	Frequency	%	Frequency	%	Frequency	%	
Water source							.567
Improved	199	58.7	328	96.8	527	77.7	
Un-improved	140	41.3	11	3.2	151	22.3	
Time take n to fetch water							.146
Source in the yard	32	9.4	31	9.1	63	9.3	
≤ 30 minutes	107	31.6	184	54.3	291	42.9	
> 30 minutes	200	59.0	124	36.6	324	47.8	
Presence of latrine							.001
No	96	28.3	107	31.6	203	29.9	
Yes	243	71.7	232	68.4	475	70.1	
Latrine type							< .0001
Improved	212	62.5	175	51.6	387	57.1	
Un-improved	127	37.5	164	48.4	291	42.9	
Utilize latrine							< .0001
No	99	29.2	133	39.2	232	34.2	
Yes	240	70.8	206	60.8	446	65.8	
Presence of feces near main house							< .0001
No	200	59.0	205	60.5	405	59.7	
Yes	139	41.0	134	39.5	273	40.3	
Solid waste disposal category							.264
Proper	185	54.6	154	45.4	339	50.0	
Improper	154	45.4	185	54.6	339	50.0	
Waste evidence near the house							< .0001
No	176	51.9	181	53.4	357	52.7	
Yes	163	48.1	158	46.6	321	47.3	
Presence of livestock							.008
No	107	31.6	81	23.9	188	27.7	
Yes	232	68.4	258	76.1	490	72.3	
Livestock place of living							< .0001
Outside the room	79	34.1	57	22.1	136	27.8	
Separate room	120	51.7	151	58.5	271	55.3	
same room with family	33	14.2	50	19.4	83	16.9	
Animal feces around the house							< .0001
No	156	46.0	213	62.8	369	54.4	
Yes	183	54.0	126	37.2	309	45.6	
Flies observed around the house							< .0001
No	182	53.7	275	81.1	457	67.4	
Yes	157	46.3	64	18.9	221	32.6	
Flies in the house							< .0001
No	218	64.3	238	70.2	456	67.3	
Yes	121	35.7	101	29.8	222	32.7	

### Prevention and control of chlamydia trachomatis infection

Almost 12.7% of children in the non-graduated district were not receiving azithromycin yet. Of children who received at least one dose of azithromycin, 8.4% of them had not received the required doses in the last two years. Of children aged 4–6 years old in the non-graduated district, only 39 (35.8%) had received three and more doses of azithromycin. Similarly, among children aged 7–9 years old, only 38 (34.2%) had received four and more doses of azithromycin. About 22.7% of mothers in graduated and 19.8% of mothers in non-graduated districts did not hear about trachoma. Nearly 28.3% of mothers in graduated and 72.9% of non-graduated districts had inadequate knowledge related to trachoma (Table 3).

**Table 3:** Prevention and control activities in mass drug administration graduated and non-graduated district, Central Gondar, Ethiopia, December 2019.

Variables	Graduated district		No-graduated district		Total		Chi-square (p-value)
	Frequency	(%)	Frequency	(%)	Frequency	(%)	
Frequency of Azithromycin doses taken							.103
No yet receiving	181	53.4	43	12.7	224	33.0	
At least one dose	158	46.6	296	87.3	454	67.0	
Time interval the child get the drug							.559
Twice in a year	-	-	7	2.4	7	1.5	
Once in a year	155	98.1	261	88.2	416	91.6	
Every two years	3	1.9	28	9.5	31	6.8	
Last time the child get the drug							.146
Six months before	1	0.6	4	1.4	5	1.1	
Nine months before	1	0.6	6	2.0	7	1.5	
Before a year	4	2.5	261	88.2	265	58.4	
Before two years	152	96.2	25	8.4	177	39.0	
Information about trachoma							.037
No	77	22.7	67	19.8	144	21.2	
Yes	262	77.3	272	80.2	534	78.8	
Knowledge about trachoma							.011
Knowledgeable	40	11.8	-	-	40	5.9	
Fairly Knowledgeable	203	59.9	92	27.1	295	43.5	
Less knowledgeable	96	28.3	247	72.9	343	50.6	

### Characteristics of children

Of 678 children, 351(51.8%) were males. Sixteen, 16 (4.7%) in graduated and 27 (8.0%) in the non-graduated district had no the habit of washing their face regularly whereas 117 (34.5%) children in graduated and 82 (24.2%) in the non-graduated district use soap for washing face regularly (Table 4).

**Table 4:** Behavioral conditions of children in mass drug administration graduated and non-graduated district, Central Gondar, Ethiopia, December 2019.

Variables	Graduated district		Non-graduated district		Total		Chi-square (p-value)
	Frequency	(%)	Frequency	(%)	Frequency	(%)	
Frequency of face washing per day							<.0001
≥ Two times	183	54.0	157	46.3	340	50.1	
One times	136	40.1	151	44.5	287	42.3	
Not washing	20	5.9	31	9.1	51	7.5	
Using soap during child face washing							<.0001
Yes, regularly	117	34.5	82	24.2	199	29.4	
Yes, some times	131	38.6	148	43.7	279	41.2	
Never	91	26.8	109	32.2	200	29.5	
Dry face after washing with towel							<.0001
Yes, regularly	63	18.6	47	13.9	110	16.2	
Yes, some times	73	21.5	36	10.6	109	16.1	
Never	203	59.9	256	75.5	459	67.7	
Ocular discharge							<.0001
No	309	91.2	298	87.9	607	89.5	
Yes	30	8.8	41	12.1	71	10.5	
Nasal discharge							<.0001
No	293	86.4	287	84.7	580	85.5	
Yes	46	13.6	52	15.3	98	14.5	
Flies on the face							<.0001
No	238	70.2	248	73.2	486	71.7	
Yes	101	29.8	91	26.8	192	28.3	
Fly-eye contacts							<.0001
No	284	83.8	284	83.8	568	83.8	
Yes	55	16.2	55	16.2	110	16.2	
Sleep in eyes							<.0001
No	273	80.5	281	82.9	554	81.7	
Yes	66	19.5	58	17.1	124	18.3	
Any dirt on face							<.0001
No	276	81.4	272	80.2	548	80.8	
Yes	63	18.6	67	19.8	130	19.2	
Child’s face condition							<.0001
Clean	218	64.3	210	61.9	428	63.1	
Unclean	121	35.7	129	38.1	250	36.9	

### Prevalence of active trachoma

The overall prevalence of active trachoma was 8.3% with 95% CI between 6.2% and 10.5% with sgnifcant variation between MDA graduated (3.5% 95% CI 1.8–5.6) and non-graduated (13% 95% CI 9.7–16.8) districts.

### Factors associated with active trachoma

In bivariate logistic regression analysis, being lived in the non-graduated district, having large family size, number of children aged 1–9 years old in the household, child space sharing, presence of latrine, type of latrine, utilization of the latrine, presence of human feces near the house, presence of livestock, presence of animal feces near a house, waste evidence near the house, fly presence around the house, presence of flies in the house, frequency of face washing per day, using soap when washing child’s face, presence of ocular and nasal discharge, flies on the child’s face, fly-eye contact, presence of sleep eye, presence of dirt on child’s face and face condition of the child were identified variables as the candidate for multivariable analysis at p-value less than 0.25. A p-value < 0.05 was used as cut off point to declare statistical significant asscoaiton with active trachoma.

In multivariable logistic regression analysis, being lived in the non-graduated district, number of children aged 1–9 years old in the household, fly presence in the house, frequency of child’s face washing habit, using soap when washing child’s face, presence of sleep in eyes and presence of dirt on the face were showed statistically significant association with active trachoma.

The odds of active trachoma among children who live in non-graduated districts were 7.39 times [AOR = 7.39, 95% CI: (3.19, 17.09)] higher compared to the graduated districts. The odds of active trachoma among children from households having flies were 3.14times higher, [AOR = 3.14, 95% CI: 1.43, 6.89)], compared to children from households without having flies. Children from households with more than two children aged 1–9 years had3.78 times higher odds of active trachoma compared to children who lived from households with 1–2 children [AOR = 3.78, 95% CI: (1.79, 7.98)].

Those children who did not wash their faces per day had6.31 times [AOR = 6.31, 95% CI: (1.81, 21.98)] higher odds of active trachoma than children who washed their face twice and more times per day. The odds of active trachoma among children who didn’t use soap during face washing were 3.34times [AOR = 3.34, 95% CI: (1.37, 8.15)] higher than children who used soap. Children who had sleep in their eyes were 3.16 times more likely to have active trachoma than children who did not have sleep in their eyes [AOR = 3.16, 95%CI: (1.42, 7.02)]. Children who had any type of dirt on their face had2.44 times, [AOR = 2.44, 95% CI: (1.08, 5.50)] higher odds of active trachoma compared to those children who had not dirty material on their face (Table 5).

**Table 5:** Bivariate and multivariable logistic regression analysis of factors associated with active trachoma (TF) among children aged 1–9 years old (n = 678), in mass drug administration graduated and non-graduated districts, Amhara Region, Ethiopia, 2019.

Variables	TF		COR(95% CI)	AOR (95% CI)
	Yes	No		
**Place where live**				
Non-graduated district	44	295	4.06 (2.11, 7.84)***	7.39 (3.19, 17.09)***
Graduated district	12	327	1	1
**Type of latrine**				
Un-improved	40	251	3.70 (2.03, 6.74)***	0.43 (0.17, 1.07)
Improved	16	371	1	1
**Presence of flies in the house**				
Yes	33	189	3.29 (1.88, 5.75)***	3.14 (1.43, 6.89)**
No	23	433	1	1
**Presence of faeces near the house**				
Yes	35	238	2.69 (1.53, 4.73)**	2.02 (0.88, 4.61)
No	21	384	1	1
**No. of children aged 1–9 years old**				
≥ 3	25	79	5.54 (3.11, 9.87)***	3.78 (1.79, 7.98)***
1–2	31	543	1	1
**Frequency of face washing per day**				
Not washing	19	32	19.59 (8.40, 45.72)	6.31 (1.81, 21.98)**
One time per day	27	260	3.43(1.63, 7.21)	2.51(0.91, 6.96)
≥ two times per day	10	330	1	1
**Use of soap**				
No	43	157	9.80 (5.13, 18.70)***	3.34(1.37, 8.15)**
Yes	13	465	1	1
**Sleep in the eye**				
Yes	34	90	9.14 (5.11, 16.33)***	3.163 (1.42, 7.02)**
No	22	532	1	1
**Any dirt on child’s face**				
Yes	33	97	7.77 (4.37, 13.80)***	2.44 (1.08, 5.50)*
No	23	525	1	1
**Child’s face condition**				
Unclean	48	202	12.48 (5.79, 26.86)***	2.73 (0.93, 7.98)
Clean	8	420	1	1

Note: Hosmer-Lemeshow test model fitness p-value was 0.816.

The variance inflation factor result was between 1 and 2.

^1^ = Reference category,

* = statistically significant at p<0.05,

** = statistically significant at p<0.01 and

*** = Statistically significant at p<0.001

## Discussion

The overall prevalence of active trachoma among children aged 1–9 years old was 8.3% [95% CI (6.2–10.5)]. This prevalence is lower than the studies conducted in different parts of Ethiopia; Madda Walabu rural district, Southeast Ethiopia (22%), Gondar Zuria district, North Gondar (12.1%), Dera district, South Gondar (15.6%), Baso Liben, West Gojjam (17.2%) and Lemo district, Hadiya Zone (13.6%) [25–29]. The prevalence is also lower than studies done in Africa Malawi (17.1%), eastern (24.9%) and western (21.7%) districts of Colombia, South America [30, 31].

Similarly, active trachoma prevalence (13%, 95% CI: 9.7%–16.8%) in MDA non-graduated districts is lower than the regional average report of trachoma impact surveys done in 2015 (25.9%) [9]. Likewise, this finding is lower than previous studies done in different parts of Ethiopia: Zala districtof Gamo Gofa Zone (36.7%); 53.9% in Ankober District, North Showa zone (53.9%); the north and South Wollo Zones (21.6%); and Gazegibela district of Wagehemra Zone (52.4%) [11, 32–34]. This might be due to the improvements in access to health services interventions given in recent years that can reduce the burden of active trachoma. For instance, successive mass drug distribution with the improvement of environmental factors has been done in the current study area which could decrease active cases. The other difference could be due to the comparative nature of this study that includes the MDA graduated district which may reduce the prevalence of active trachoma in this study.

However, the overall prevalence is higher than the WHO trachoma elimination target (a prevalence of active trachoma (TF) in children aged 1–9 years of old < 5%) [21]. Even though it was planned to eliminate the disease by the year 2020, finding of this study calls the implementation of A, F, and E components of SAFE are mandatory till the prevalence meets the expected target. Similarly, the overall prevalence is higher than Global Trachoma Mapping Project studies before mass antibiotic initiation began in seven districts of the Republic of the Congo (TF prevalence was 2.5%) and in Benue State, Nigeria (TF prevalence was 0.3% to 5.3%) [35, 36]. The later studies done in Congo and Nigeria were done as a baseline survey for mapping the diseases to initiate SAFE interventions. However, this study was done after successive SAFE interventions in the current study area. Therefore, the difference might be due to the level of variations in the endemicity of the study areas.

The prevalence of active trachoma among children aged 1–9 years old in the MDA graduated district was 3.5%, (95% CI: 1.8–5.6) which is in line with WHO recommendations of TF (5%) at the district level [21]. Besides, the finding of this study is consistent with the studies done in two regions of Mali reported TF prevalence of 1.7% (0.8%–2.6%) in Ouelessebougou, 2.9% (1.5%–4.2%) in Nara, and 2.5% (0.9%–4.1%) in Kita districts. However, it is lower than the reported TF prevalence of 11.4% (9.1%–13.7%) in Koulikoro, 14.1% (9.2%–19.0%) in Kolokani, and 15.4% (11.7%–19.0%) in Bafoulabe districts in the same study [37]. This might be due to trachoma endemicity at baseline which is predictive of return of infection after the antibiotic intervention [38].

This study revealed that the prevalence of active trachoma among children aged 1–9 years old had shown statistically significant differences between MDA graduated and non-graduated districts. The possible explanation for this difference might be due to the lower antibiotic coverage in non-graduated districts than what is recommended by the World Health Organization that is at least 80% of the population should receive antibiotics for eliminations of trachoma. This is confirmed by a previous study done in the Amhara region which reported administrative reports of the MDA campaign were higher than the population-based self-reported coverage of antibiotic supplementations [39].

This study also revealed similar discrepancies, for instance, children with the age of 4–6 years old in the non-graduated district are expected to receive at least three doses. However, only 39 (35.8%) of them in this age group had received three doses and above. Likewise, among children aged 7–9 years old, only 38 (34.2%) of them had received four doses and above. Therefore, children and mothers who have intimate contact with children missed during mass drug administration campaigns may leave them untreated which then reintroduce infection into treated communities.

The other possible explanation for this difference might be related to the knowledge of mothers/caretakers on signs and symptoms, mode of transmission, and prevention methods of trachoma. Almost twelve percent (11.8%) of mothers/caretakers in graduated districts have good knowledge about trachoma compared to mothers/caretakers of children in the non-graduated district. This is very important to have a good practice of children’s behavioral factors. For instance, 34.5% of mothers use soap regularly for washing their children’s faces in MDA graduated district compared to 24.2% of mothers in the MDA non-graduated district.

In this study, being a child from households with flies in the house was a significant predictor of active trachoma. This might be due to the increased access of *Musca sorbens* flies that promotes the risk of transmission of active trachoma which is supported by a previous study done in North Gondar, Ethiopia [26].

In this study, children from households with more than two children aged 1–9 years old had also higher active trachoma. This finding is in line with results from cross-sectional studies conducted in Gonji Kollela district, West Gojjam Zone, and Zala district, Gamo Gofa Zone, of Ethiopia [10, 32]. The possible explanation for this association might be mother/caretakers are responsible for the caretaking of children, as the number of children in the household increases, it is difficult to get take care of all children and the possibility of sharing fomites enables the exchange of secretions and promotes infection with the causative agent.

Sleep in the child’s eyes and the presence of dirt on a child’s face were also a significant predictor of active trachoma. The possible explanation might be the sleep in the eye and the presence of dirt on a child’s face is observed when children continued without washing their faces for numbers of days. This is supported by the current study and other studies conducted in Ethiopia as those children who had not washed their faces daily were more likely to have active trachoma than children who washed twice and more times per day [26, 34]. This might be due to frequent face washing habit improves the facial cleanliness of children and thereby their face did not receive the vector, eye seeking flies, responsible for the transmission of the causative agent.

Like other studies in Ethiopia in Woliso town and Baso Liben districts [27, 40], the use of soap for washing the face was identified as a predictor of active trachoma. Some studies show the prevalence of active trachoma is higher in children who do not use soap compared to children who use soap. Soap utilization while washing the child’s face is a key factor to effectively remove the causative agent discharged with ocular secretions. This interrupts transmission pathways from the reservoir to healthy children.

## Limitations

Although comparative community based of this study in graduated and non-graduated districts could be the strengths of study; there might be the possibility of social desirability bias such as frequency of washing a child face per day, use of soap, and the possibility of recall bias among respondents answering questions relating to events happening in the past, such as frequency of azithromycin doses the child received in the past years.

## Conclusions

Prevalence of active trachoma (TF) among children aged 1–9 years old was high in the study area and had shown a significant variation between MDA graduated and non-graduated districts. The result showed that the prevalence of active trachoma (TF) in the MDA graduated district was below the WHO threshold of 5% to determine trachoma. However, in MDA non-graduated district it is far from the elimination target of trachoma. Living in a non-graduated district, fly presence in the house, being a child from households with more than two children aged 1–9 years old, did not wash face per day, did not use soap while washing face and the presence of sleep in eyes and presence of any dirt on the face were identified as significant predictors of active trachoma (TF) among children aged 1–9 years old. Mass drug administration should be strengthened to reduce the burden of active trachoma. Promotion of identified modifiable behavioral factors such as health education programs for the communities about prevention and control of active trachoma through fly control and keeping facial cleanliness by washing their child’s face with soap are the areas of intervention in reducing active trachoma. The promotion of the utilization of family planning to reduce the number of children aged 1–9 years old in a household is important.

## Supporting information

S1 Dataset (SAV)

S1 File (DOCX)

## Abbreviations

AOR: Adjusted Odds Ratio
CI: Confidence Interval
COR: Crude Odds Ratio
GET: Global Alliance for Trachoma Elimination
HH: House Hold
MDA: Mass Drug Administration
SAFE: Surgery, Antibiotic, Facial cleanliness, and Environmental improvements
SD: Standard Deviation
SPSS: Statistical packages for Social Sciences
TF: Trachomatous Inflammation Follicular
TT: Trachomatous Trichiasis
WASH: Water, Sanitation and Hygiene
WHO: World Health Organization
