# Supplementary material for: Prevalence and associated factors of active trachoma among children aged 1-9 years old in mass drug administration graduated and non-graduated districts in Northwest Amhara region, Ethiopia: A comparative cross-sectional study
Source: PLoS One. 2020 Dec 15;15(12):e0243863. doi: 10.1371/journal.pone.0243863 (PMC7737887; doi:10.1371/journal.pone.0243863)
Supplement: S1 File — (DOCX) [file pone.0243863.s002.docx]

English Questionnaire

Participant ID No___________ Kebele ____ Interviewer name_________________________ Date of interview____________ Starting time _____________Ending time ___________

Please encircle the correct answer which to indicate number and write a correct number example age put in a year.

**Part I: Socio-demographic characteristics**

| **S/No** | **Questions** | **Answer** | Code | **Skip** |
| --- | --- | --- | --- | --- |
| 101 | Age of the child | _________ |  |  |
| 102 | Sex of the child | Male  Female | 1  2 |  |
| 103 | Level of education of the child | Not yet attended  Attended | 1  2 |  |
| 104 | Age of the mother/caregiver(years) | _________ |  |  |
| 105 | What is your relationship with the children? | Parent  Guardian | 1  2 |  |
| 106 | Religion of mother/caregiver | Orthodox  Muslim  protestant  catholic  Others____________ | 1  2  3  4  99 |  |
| 107 | Level of education of mother/caregiver | Unable to read & write  Read and write  Primary level  Secondary level  Diploma and above | 1  2  3  4  5 |  |
| 108 | Maternal occupation | Unemployed  Civil servant  Daily laborer  Merchant  house wife  Farmer  Other(specify)________________ | 1  2  3  4  5  6  99 |  |
| 109 | Marital status of mother/caregiver | Single  Married  Divorced  Widowed  lives separately | 1  2  3  4  5 |  |
| 110 | If you have a husband, what is the educational level of your husband? | Unable to read & write  Read and write  Primary level  Secondary level  Diploma and above | 1  2  3  4  5 |  |
| 111 | If you have a husband, what is the occupational status of your husband? | Unemployed  Civil servant  Daily laborer  Merchant  Farmer  Other specify_______________ | 1  2  3  4  5  99 |  |
| 112 | Total number of family size | _______________ |  |  |
| 113 | Total number of <10 years children | _______________ |  |  |
| 114 | Do children share the same sleeping space or bedding? | Yes  No | 1  2 |  |
| 115 | Place of residence | Urban  Rural | 1  2 |  |

**Wealth index (Ownership of durable assets and housing characteristics)**

| 116 | Where do you live? | Rented house  Own house | 1  2 |  |
| --- | --- | --- | --- | --- |
| 117 | Number of rooms in the dwelling place | ________________ |  |  |
| 120 | How many bed rooms in the house | ________________ |  |  |
| 124 | Where do you get drinking water? | Piped into the yard/plot water  Piped to neighbor  Hand pumps  Public tap/standpipe  Protected well/spring  Rainwater  Unprotected well/spring  Surface water (river/dam/ /pond/stream/canal/ irrigation channel)  Other list____________________ | 1  2  3  4  5  6  7  8  99 |  |
| 125 | Do you have latrine for the household members? | Yes  No | 1  2 |  |
| 126 | Observe that which type of latrine the household have? | pour flush latrine  Ventilated improved pit latrine (VIP)  Pit latrine with slab  Pit latrine without slab/open pit  No facilities or bush or field  Other (specify )________________ | 1  2  3  4  5  99 |  |
| Among the following materials, which one do you own? (more than one answer is possible )(measuring of Household Economic status ) | | |  |  |
| 127 | Watch | Yes  No | 1  0 |  |
| 128 | Sofa | Yes  No | 1  0 |  |
| 129 | Chair | Yes  No | 1  0 |  |
| 130 | Table | Yes  No | 1  0 |  |
| 131 | Bed and mattress which made from cotton spring | Yes  No | 1  0 |  |
| 132 | Horse’s Cart | Yes  No | 1  0 |  |
| 133 | Radio | Yes  No | 1  0 |  |
| 134 | Television | Yes  No | 1  0 |  |
| 135 | House phone | Yes  No | 1  0 |  |
| 136 | Fridge | Yes  No | 1  0 |  |
| 137 | Mobile | Yes  No | 1  0 |  |
| 137 | Cycle | Yes  No | 1  0 |  |
| 138 | Motor cycle | Yes  No | 1  0 |  |
| 139 | Bajaj/car | Yes  No | 1  0 |  |
| 140 | Have you bank account | Yes  No | 1  2 |  |
| 141 | If others (specify)_______________________ |  | 99 |  |
| 142 | Do you have your own farm for the purpose of agriculture/cropping? | Yes  No | 1  0 |  |
| 143 | If the answer for Q#142 is yes, how many in hectare? | __________hectare(1 hectare = 4 timad) |  |  |
|  | From the following household animal do you have? (can answer more than one) | |  | Number |
| 144 | Ox/ cow | Yes  No | 1  0 | __________ |
| 145 | Horse/donkey/ mule | Yes  No | 1  0 | __________ |
| 146 | Goat | Yes  No | 1  0 | __________ |
| 147 | Sheep | Yes  No | 1  0 | __________ |
| 148 | Hen | Yes  No | 1  0 | __________ |
| 149 | Beehive | Yes  No | 1  0 | __________ |
| 150 | Others(specify)_______________________ |  | 99 | __________ |

**Part II: Household Level Environmental factors**

| **S/No** | **Questions** | **Answer** | Code | **skip** |
| --- | --- | --- | --- | --- |
| 201 | Do you have livestock? | Yes  No | 1  0 |  |
| 202 | If you have livestock, where do they live? | Outside the room/‟beret laye‟  Separate room for themselves  In the same room with family | 1  2  3 |  |
| 203 | Are there animal feces observed around the house? | Yes  No | 1  0 |  |
| 204 | Are there flies in the house? | Yes  No | 1  0 |  |
| 205 | Flies observed around the house | Yes  No | 1  0 |  |
| About water supply conditions | | Answer | code | skip |
| 206 | What is your common water collection material? | 10 litre jerrycan/pot  15 litre jerrycan/pot  20 litre jerrycan/pot  25 litre jerrycan/pot  Other specify ___________________ | 1  2  3  4  5 |  |
| 207 | How many times you collect water per day by this collection material? | **___________________** |  |  |
| 208 | On average how much litter of water do your family use per day | **_____________________** |  |  |
| 209 | How long does it take to go there, get water, and come back? | Water source in the yard  Less than 30 minutes  Greater than 30 minutes | 1  2  3 |  |
| About latrine conditions | | Answer | code | skip |
| 210 | Where do you and other adults in the household usually defecate? | Private latrine  Shared latrine  No structure, outside near the house  No structure, in the bush or field  Other(specify)______________ | 1  2  3  4  99 |  |
| 211 | Observe the presence of feces near the vicinity of the main house | Yes  No | 1  0 |  |
| 212 | What method do you use to dispose of the solid waste which is generated in the house? | Throwing near the house /open field/  In the pit prepared for solid waste  burning near the yard  collected by municipality  Other(specify)______________ | 1  2  3  4  99 |  |
| 213 | Is there evidence of solid waste or garbage within 20 meters of the house ( This does not include animal droppings) | Yes  No | 1  0 |  |

Part III. Risk Factors for Chlamydia Trachomatis Infection

| **S/No** | **Questions** | | **Answer** | **Code** | **Skip** |
| --- | --- | --- | --- | --- | --- |
| 301 | How many times the child has received azithromycin? | | Not received yet  One time  Two times  Three times  Four and more times | 1  2  3  4  5 |  |
| 302 | If the child has received azithromycin,by what time interval did he/she get the drug? | | Once in a year  Two times in a year  Every two years | 1  2  3 |  |
| 303 | If the child has received azithromycin, when he/she get the drug for the last time? | | Three months before  Six months before  Nine months before  Before a year  Before two years | 1  2  3  4  5 |  |
| **Health Education about Trachoma** | | | |  |  |
| 304 | Have you ever informed about trachoma? | | Yes  No | 1  0 |  |
| 305 | If you say” yes” for Q # 304 from which source do you hear?**( more than one answer is possible)** | | Health personnel  Health extension workers  Media/radio, TV  From peers, Community  Others/Specify________________ | 1  2  3  4  99 |  |
| **Knowledge about Trachoma** | | | |  |  |
| 306 | What are the sign & symptoms of trachoma disease?**( more than one answer is possible)** | No Sign and Symptom  Burning  Itching Photophobia  Ocular discharge  Foreign body sensation  Red eye  I don’t know | | 1  2  3  4  5  6  7  8 |  |
| 307 | Where people have got trachoma from? **( more than one answer is possible)** | From diseased people  From animals  From dirt  Others/specify____________ | | 1  2  3  99 |  |
| 308 | How trachoma is transmitted from person to person?**( more than one answer is possible)** | By flies  By sharing towels  By sharing bed sheets  By sharing eye makeup  By close contact of body  By touching your eyes by unwashed hands I don’t know | | 1  2  3  4  5  6  7 |  |
| 309 | How do we prevent trachoma? **( more than one answer is possible)** | Taking medicine/drugs  Keeping personal hygiene  Proper use of latrine  Making the compound clean  I don’t know  Others/Specify________________ | | 1  2  3  4  5  99 |  |

Part IV. Child’s behavioral factors and status of Active trachoma

| **S/No** | **Questions** | **Answer** | | Code | **skip** | |
| --- | --- | --- | --- | --- | --- | --- |
| 401 | Do you wash your child’s face regularly? | Yes  No | | 1  0 |  | |
| 402 | If you yes for Q 401, How many times do you wash child’s face per day? | Once  Twice  More than twice  Don’t know | | 1  2  3  4 |  | |
| 403 | Do you use soap when you are washing your child’s face? | Yes, regularly/always  Yes, sometimes  Never | | 1  2  3 |  | |
| 404 | If you say Q403”# 3” what is the reason? | Unavailability/ unable to purchase the soap Ignorance of the importance of using soap I don’t know  Any other mention___________________ | | 1  2  3  99 |  | |
| 405 | Do you dry your child’s face after washing with towel? | Yes, regularly  Yes,some times  Never | | 1  2  3 |  | |
|  | Facial cleanliness observation |  | |  |  | |
| 406 | Ocular discharge | Yes  No | | 1  0 |  | |
| 407 | Nasal discharge | Yes  No | | 1  0 |  | |
| 408 | Flies on the face | Yes  No | | 1  0 |  | |
| 409 | Fly-eye contacts | Yes  No | | 1  0 |  | |
| 410 | Sleep in eyes | Yes  No | | 1  0 |  | |
| 411 | Any dirt on face | Yes  No | | 1  0 |  | |
| 412 | Clean face | Yes  No | | 1  0 |  | |
| 413 | Other/ specify__________________ |  | | 99 |  | |
| **Eye examination** | | | | | | |
| 414 | Is there the TF/Active trachoma / sign present in the eye? (filled by physical examination of two eyes of the child) | | Sign absent  Sign present  Not able to grade | 0  1  2 | |  |

Thank you for your valuable information and participation!!!

የአማርኛመጠይቅ

የተሳታፊመለያቁጥር______ ቀበሌ______ የቃለመጠይቅአድራጊውስም___________________ ቀን____________ የተጀመረበትሰዓት____________ የተጠናቀቀበትሰዓት ________________

**ክፍል 1፡የሶሽዮ-ዲሞግራፉመረጃመጠይቅ**

| ተ/ቁ | ጥያቄ | አማራጭ | ኮድ | እለፍ |
| --- | --- | --- | --- | --- |
| 101 | የሕፃኑ/ኗዕድሜበአመት | _________________ |  |  |
| 102 | የሕፃኑ/ኗፆታ | ወንድ  ሴት |  |  |
| 103 | የህፃኑ/ኗየትምህርትደረጃ | ትምህርትያልጀመረ/ች  ትምህርት የጀመረ/ች | 1  2 |  |
| 104 | የሕፃኑ/ኗእናት/አሳዳጊዕድሜበአመት | _________________ | 1  2 |  |
| 105 | ከህፃኑ/ከህፃኗ ጋር ያለወት ዝምድና | የስጋ ወላጅ  የህፃኑ ተንከባካቢ | 1  2 |  |
| 106 | የቤተሰብወይምያአሳዳጊሀይማኖት | ኦርቶዶክስ  ሙስሊም  ፕሮቴስታንት  ካቶሊክ  ሌላካለይገለፅ______________ | 1  2  3  4  99 |  |
| 107 | የሕፃኑ/ኗእናት/አሳዳጊየትምህርትደረጃ | ማንበብናመፃፍየማትችል  ማንበብእናመፃፍየምትችል  አንደኛደረጃ  ሁለተኛደረጃ  ኮሌጂእናዩንቨርስቲ | 1  2  3  4  5 |  |
| 108 | የሕፃኑ/ኗእናት/አሳዳጊየስራሁኔታ | ስራየሌላት  የመንግስትሰራተኛ  የቀንሰራተኛ  ነጋዴ  የቤትእመቤት  ገበሬ  ሌላካለይገለፅ______________ | 1  2  3  4  5  6  99 |  |
| 109 | የሕፃኑ/ኗእናት/አሳዳጊየጋብቻሁኔታ | ያላገባች  ያገባች  የፈታች  ባልየሞተባት  ተለያይተው የሚኖሩ | 1  2  3  4  5 |  |
| 110 | ባለቤትካለዎትየባለቤትዎየትምህርትደረጃ | ማንበብናመፃፍየማይችል  ማንበብእናመፃፍየሚችል  አንደኛደረጃ  ሁለተኛደረጃ  ዩንቨርስቲእናኮሌጂ | 1  2  3  4  5 |  |
| 111 | ባለቤትካለዎትየባለቤትዎየስራሁኔታ | ስራየሌለው  የመንግስትሰራተኛ  የቀንሰራተኛ  ነጋዴ  ገበሬ  ሌላካለይገለፅ_____________ | 1  2  3  4  5  99 |  |
| 112 | ጠቅላላየቤተሰብቁጥርብዛት | ________________ |  |  |
| 113 | ጠቅላላከ10 አመትበታችህፃናትብዛት | ________________ |  |  |
| 114 | ከ10 አመትበታችህፃናትየመኝታክፍሎችንወይምየመኝታአልጋዎችንበጋራይጠቀማሉ? | አዎን  የለም | 1  2 |  |
| 115 | የመኖሪያ ቦታ | ከተማ  ገጠር | 1  2 |  |

**የሀብትሁኔታጠቋሚመረጃ**

| ተ/ቁ | ጥያቄ | አማራጭ | ኮድ | እለፍ |
| --- | --- | --- | --- | --- |
| 116 | የትነውየሚኖሩት? | ከራሳችንቤት  ከኪራይቤት | 1  2 |  |
| 117 | የሚኖሩበትቤትስንትክፍሎችአሉት? | ________________ |  |  |
| 120 | ቤቱስንትምኝታክፍልአለዉ? | ________________ |  |  |
| 124 | የቤተሰብዎአባላትበዋናነትየሚጠቀመውየውኃመገኛየትኛውነው? | በግቢውስጥያለየቧንቧውኃ  ከጎረቤትያለየቧንቧውኃ  በእጅየሚነቀነቅየጋራዉሃ  ቦኖዉሃ  የተከለለየጉድጋድ /የምንጭዉሃ  የዝናብውኃ  ያልተከለለየጉድጋድ/የምንጭዉሃ  የምንጭ /የወራጅ/ ኩሬየግድብዉሃ  ሌላካለይገለፅ_______________ | 1  2  3  4  5  6  7  8  99 |  |
| 125 | ለቤተሰብዎየሚያገለግልየመፀዳጃቤትአለ? | አዎን  የለም | 1  2 |  |
| 126 | የመጸዳጃቤቱምንአይነትአንደሆነበምልከታይረጋገጥ | በዉሃየሚሰራሽንትቤት  የአየርማስወጫቱቦያለዉሽንትቤት  ርብራብየሌለውባህላዊሽንትቤት  ርብራብያለውባህላዊሽንትቤት  ሽንትቤትየለም/ቁጥቋጦ/ሜዳላይ  ሌላካለይገለፅ___________ | 1  2  3  4  5  99 |  |
|  | ክዚህበታችከተዘረዘሩትንብረቶችበቤታችሁዉስጥያላችሁየቱነዉ? (ከአንድበላይመመለስይቻላል)(የበተሰቡን የገቢ ሁኔታ መለካት) | አማራጭ | ኮድ | እለፍ |
| 127 | ሰአት | አዎን  የለም | 1  0 |  |
| 128 | ሶፋ | አዎን  የለም | 1  0 |  |
| 129 | ወንበር | አዎን  የለም | 1  0 |  |
| 130 | ጠረንጴዛ | አዎን  የለም | 1  0 |  |
| 131 | የጥጥ/የእስቦንጅ / አስፕሪንግፍራሽያለዉአልጋ | አዎን  የለም | 1  0 |  |
| 132 | የፈረስጋሪ | አዎን  የለም | 1  0 |  |
| 133 | ሬድዮ | አዎን  የለም | 1  0 |  |
| 134 | ቴሌቪዥን | አዎን  የለም | 1  0 |  |
| 135 | የቤትስልክ | አዎን  የለም | 1  0 |  |
| 136 | ፍሪጅ | አዎን  የለም | 1  0 |  |
| 137 | ሞባይልስልክ | አዎን  የለም | 1  0 |  |
| 137 | ሳይክል | አዎን  የለም | 1  0 |  |
| 138 | ሞተርሳይክል | አዎን  የለም | 1  0 |  |
| 139 | ባጃጅ/መኪና | አዎን  የለም | 1  0 |  |
| 140 | የባንክቡክ | አዎን  የለም | 1  0 |  |
| 141 | ሌላካለይጥቀሱ _________________________ |  | 99 |  |
| 142 | የግላችሁየሆነለምርት/እርሻየሚሆንመሬትአላችሁ  ወይ ? | አዎን  የለም | 1  0 |  |
| 143 | ለጥያቄ ቁጥር 142 የተሰጠው መልስ አዎን ከሆነ የመሬቱ ብዛት ምን ያህል ይሆናል? | ____________ ሄክታር /1ሄ = 4 ጥማድ/ |  |  |
|  | ከሚከተሉትየቤትእንሰሳትዉሰጥየትኛውአላችሁ? | አማራጭ | ኮድ | ብዛት በቁጥር |
| 144 | በሬ፣ላም | አዎን  የለም | 1  0 | __________ |
| 145 | ፈረስ/አህያ፣በቅሎ | አዎን  የለም | 1  0 |  |
| 146 | ፍየል | አዎን  የለም | 1  0 | __________ |
| 147 | በግ | አዎን  የለም | 1  0 |  |
| 148 | ዶሮ | አዎን  የለም | 1  0 | __________ |
| 149 | የንብቀፎ | አዎን  የለም | 1  0 | __________ |
| 150 | ሌላካለይጥቀሱ __________________________ |  | 99 | __________ |

**ክፍል2: የቤትእናየአካባቢሁኔታ**

| ተ/ቁ | ጥያቄዎች | አማራጭ | ኮድ | እለፍ |
| --- | --- | --- | --- | --- |
| 201 | የቤትእንስሳትአላችሁወይ? | አወን  የለም | 1  0 |  |
| 202 | የቤትእንስሳትካሏችሁየትነዉየሚያድሩት? | ዉጭላይ/በረትላይ  የተለየክፍልማደሪያአላቸዉ  ከቤተሰቡጋርበአንድክፍል | 1  2  3 |  |
| 203 | በመኖሪያቤቱዙሪያየሚታይየእንስሳትፅዳጅአለወይ ? | አወን  የለም | 1  0 |  |
| 204 | በመኖሪያቤቱዙሪያየሚታዩዝንቦችአሉወይ ? | አወን  የለም | 1  0 |  |
| 205 | በመኖሪያቤቱውስጥየሚታዩዝንቦችአሉወይ ? | አወን  የለም | 1  0 |  |
|  | የውኃአቅርቦትንበተመለከተ | አማራጭ | ኮድ | እለፍ |
| 206 | ብዙ ጊዜ ውኃ የሚቀዱት በምንድን ነው? | 10 ሊትር በሚይዝ ጀሪካን/ማሰሮ  15 ሊትር በሚይዝ ጀሪካን/ማሰሮ  20 ሊትር በሚይዝ ጀሪካን/ማሰሮ  25 ሊትር በሚይዝ ጀሪካን/ማሰሮ  ሌላ ካለ ይገለጽ________________ | 1  2  3  4  5 |  |
| 207 | በዚህ የውኃ መቅጃ እቃ ለሁሉም አይነት አገልግሎት በቀን ምን ያህል ጊዜ ይቀዳሉ? | **____________**ጀሪካን/ማሰሮ |  |  |
| 208 | ቤተስቡበቀንበአማካይ ምንያህልሊትርውኃይጠቀማል? | _________________ |  |  |
| 209 | የሚጠቀሙትን ውኃ ወደመገኛውሂዶቀድቶለመመለስምንያህልጊዜይጨርሳል? | የውኃመገኛውበግቢውስጥነው  < 30 ደቂቃይጨርሳል  > 30 ደቂቃይጨርሳል | 1  2  3 |  |
|  | የመጸዳጃቤትንበተመለከተ | አማራጭ | ኮድ | እለፍ |
| 210 | እርስዎናጎልማሳየሆኑየቤተሰብዎአባላትየምትጸዳዱትየትነው? | በግልመጸዳጃቤት  በጋራመጸዳጃቤት  መጸዳጃቤትየለም፣ውጭላይቤትአጠገብ  መጸዳጃቤትየለም፣ቁጥቋጦውስጥ/ ሜዳለይ  ሌላካለይገለፅ_______________ | 1  2  3  4  99 |  |
| 211 | በመኖሪያቤቱዙሪያየሰዎችፅዳጅመኖሩበምልከታይረጋገጥ | አወን  የለም | 1  0 |  |
| 212 | ከመኖሪያቤትዎየሚፈጠረውንደረቅቆሻሻበየትኛውመንገድያሰወግዳሉ? | ቤቱአቅራቢያውጭላይወይምሜዳላይበመወርወር  በተዘጋጀየደረቅቆሻሻጉደጓድ  ግቢውስጥበማቃጠል  በመዘጋጃቤትይሰበሰባል  ሌላካለይገለፅ___________ | 1  2  3  4  99 |  |
| 213 | ከዋናውመኖሪያቤትበ 20 ሜትርርቀትላይየደረቅቆሻሻመኖሩይረጋገጥ | አወን  የለም | 1  0 |  |

**ክፍል 3፡- የአፍላትራኮማብክለትንለመከላከልእየተሰሩያሉስራዎች**

| ተ/ቁ | ጥያቄዎች | | አማራጭ | ኮድ | እለፍ |
| --- | --- | --- | --- | --- | --- |
| 301 | የአፍላትራኮማልየታየተደረገለትህፃንዎየአፍላትራኮማመከላከያመድኃኒትስንትጊዜ ወስዷል? | | ወስዶአያውቅም  አንድጊዜ  ሁለትጊዜ  ሶስትጊዜ  አራትጊዜእናከዚያበላይ | 1  2  3  4  5 |  |
| 302 | የአፍላትራኮማልየታየተደረገለትህፃንዎየአፍላትራኮማመከላከያመድኃኒት ወስዶ/ዳ የሚያውቅ ከሆነ በስንት ጊዜ ልዩነት ነው የወሰደው/ችው? | | በአመት ሁለት ጊዜ  በአመት አንድ ጊዜ  በሁለት አመት አንድ ጊዜ | 1  2  3 |  |
| 303 | የአፍላትራኮማልየታየተደረገለትህፃንዎየአፍላትራኮማመከላከያመድኃኒት ወስዶ/ዳ የሚያውቅ ከሆነ ለመጨረሻ ጊዜ የወሰደው/ችው መቸ ነው? | | ከሶስት ወር በፊት  ከስድስት ወር በፊት  ከዘጠኝ ወር በፊት  ከአንድ አመት በፊት  ከሁለት በፊት | 1  2  3  4  5 |  |
| ስለትራኮማበሽታየተሰጠየጤናትምህርት | | | |  |  |
| 304 | ስለትራኮማበሽታምንነትየጤናትምህርትአግኘተውያውቃሉ? | | አወን  የለም | 1  0 |  |
| 305 | ለጥያቄቁጥር 304መልስዎአዎንከሆነ፣የጤናትምህርቱየተሰጠውበማንነው?  **ከአንድ በላይ መልስ መመለስ ይቻላል** | | በጤናባለሙያ  በጤናኤክሰቴንሽንባለሙያ በሚዲያ/በራዲዮ/በቴሌቭዥን  ከአቻዎቸ/ከጎረቤቶቸ  ሌላካለይገለፅ________________ | 1  2  3  4  99 |  |
| ሰለትራኮማበሽታያላቸውእውቀት | | | |  |  |
| 306 | የትራኮማበሽታምንምንምልክቶችአሉት? **ከአንድ በላይ መልስ መመለስ ይቻላል** | ምንምምልክትየለውም  ማቃጠል  ማሳከክ የብርሃንጨረርንመፍራት  ከአይንየሚወጣፈሳሽነገርመኖር  ወደአይንባዕድነገርአንደገባአይነትሰሜት/መቆርቆር  የአይንመቅላት  አላውቅም | | 1  2  3  4  5  6  7  8 |  |
| 307 | ሰዎችየትራኮማበሽታንከየትያገኙታል?  **ከአንድ በላይ መልስ መመለስ ይቻላል** | ከታመሙሰዎቸች  ከእንስሳት  ከቆሻሻ  ሌላካለይገለፅ____________ | | 1  2  3  99 |  |
| 308 | የትራኮማበሽታከሰውወደሰውእንዴትይተላለፋል? **ከአንድ በላይ መልስ መመለስ ይቻላል** | በዝንቦችአማካኝነት  የፊትማድረቂያፎጣንበጋራበመጠቀም  የአልጋልብሶችንበጋራበመጠቀም  የአይንመዋቢያአቃዎችንበጋራበመጠቀም  በሰውነትንክኪ  ንፅህናውባለተጠበቀእጅአይንንበመንካት  አላውቅም | | 1  2  3  4  5  6  7 |  |
| 309 | የትራኮማበሽታንእንዴትመከላከልይቻላል? **ከአንድ በላይ መልስ መመለስ ይቻላል** | መድኃኒትወይምከኒንበመውሰድ  የግልንፅሀናንበመጠበቅ  ሁለጊዜመጸዳጃቤትበመጠቀም  የአካባቢያችንንንጽህናበመጠበቅ  አላውቅም  ሌላካለይገለፅ____________ | | 1  2  3  4  5  99 |  |

**ክፍል 4፡- የህፃናትየንፅህናእናየአፍላትራኮማሁኔታ**

| ተ/ቁ | ጥያቄዎች | አማራጭ | ኮድ | እለፍ |
| --- | --- | --- | --- | --- |
| 401 | የልጆችዎንፊትዘወትርያጥባሉ? | አወን  የለም | 1  0 |  |
| 402 | ለጥያቄ ቁጥር 401 መለስዎ አዎን ከሆነ የልጆችዎንፊትበቀንምንያህልጊዜያጥባሉ? | አንድጊዜ  ሁለትጊዜ  ከሁለትጊዜበላይ  አላወቀውም | 1  2  3  4 |  |
| 403 | የልጆችዎንፊትበሚያጥቡበትወቅትሳሙናይጠቀማሉ? | አወንዘወትር/ሁልጊዜ  አወንአልፎአልፎ  አልጠቀምም | 1  2  3 |  |
| 404 | ለጥያቄቁጥር 403 መልስዎ 3 ከሆነየልጆችዎንፊትበሚያጥቡበትወቅትሳሙናየማይጠቀሙበትምክንያትምንድንነው? | ስሌለኝ/ ሳሙናመግዛትስለማልችል  በሳሙናፊትንመታጠብጥቅምስለሌለው  አላውቅም  ሌላካለይገለጽ___________ | 1  2  3  99 |  |
| 405 | የልጆችዎንፊትካጠቡበኃላበፎጣያደርቃሉ? | አወን፣ዘወትር  አወን፣አልፎአልፎ  አልጠቀምም | 1  2  3 |  |
| የህፃኑ/ኗየፊትንጽህናሁኔታምንይመስላል?በምልከታይረጋገጥ | | | ኮድ |  |
| 406 | ከህፃኑ/ኗአይንየሚወጣፈሳሽአለ? | አለ  የለም | 1  0 |  |
| 407 | ከህፃኑ/ኗአፍንጫየሚወጣፈሳሽአለ? | አለ  የለም | 1  0 |  |
| 408 | ከህፃኑ/ኗፊትዝንቦችአለ? | አለ  የለም | 1  0 |  |
| 409 | ከህፃኑ/ኗአይንዝንብአለ? | አለ  የለም | 1  0 |  |
| 410 | ከህፃኑ/ኗአይንላይቅምጥአለ? | አለ  የለም | 1  0 |  |
| 411 | ከህፃኑ/ኗፊትላይየሚታይቆሻሻአለ? | አለ  የለም | 1  0 |  |
| 412 | የህፃኑ/ኗፊትንጽህናውየተጠበቀነውን? | አለ  የለም | 1  0 |  |
| 413 | ሌላካለይገለፅ_____________ |  | 99 |  |
| የህፃኑ/ኗአይንየአፍላትራኮማልየታውጤት | | አማራጭ | ኮድ | እለፍ |
| 414 | የህፃኑ/ኗአይንየላይኛውቆብላይየአፍላትራኮማምልከትአለወይ?**(ሁለቱንም አይኖችን በማየት ይሞላ)** | የአፍላትራኮማምልከትየለም  የአፍላትራኮማምልከትአለ  የአፍላትራኮማለመለየትያስቸግራል | 0  1  2 |  |

**ስለሰጡኝጠቃሚመረጃናስለተሳትፎዎከልብአመሰግናለሁ!!!!**
